# Differences in Plasma Cytokine Levels between Elite Kayakers and Nonathletes

**DOI:** 10.1155/2013/370354

**Published:** 2013-05-27

**Authors:** G. F. Borges, L. Rama, S. Pedreiro, F. Alves, A. Santos, A. Massart, A. Paiva, A. M. Teixeira

**Affiliations:** ^1^Center for Sports and Physical Activity, Sport Sciences and Physical Education School, University of Coimbra, 3040-156 Coimbra, Portugal; ^2^Health and Biotechnology Institute, Federal University of Amazonas, 69460-000 Coari, AM, Brazil; ^3^Center of Histocompatibility, 3001-301 Coimbra, Portugal; ^4^Human Kinetics School, Technical University of Lisbon, 1499-002 Lisbon, Portugal; ^5^Estádio Universitário, Pavilhão 3, Sta. Clara, 3040-156 Coimbra, Portugal

## Abstract

Regular moderate exercise has been shown to have anti-inflammatory effects that help prevent several chronic diseases. However, the effects of chronic training an elite athletes have not been the focus of much research. This study aimed to determine whether there were differences in cytokine levels (IL-1**β**, IL-1ra, IL-6, IL-10, IL-18, IFN-**γ**, and TNF-**α**) in circulating peripheral blood (PB) between elite kayakers and nonathletes. Subjects were 13 elite male kayakers, aged 20.0 ± 3 years, with average body mass of 75.0 ± 7.9 kg and 177.3 ± 7.1 cm height and with a VO_2max_ of 58.3 ± 7.8 mL·kg^−1^·min^−1^. The nonathletes were 7 men, aged 18.2 ± 1.1 years, body mass of 81.3 ± 13.8 kg, and 171.9 ± 4.5 cm height. Blood samples were collected after six weeks of offtraining and before the start of a new training season. PB leukocyte populations were determined by flow cytometry. Cytokine levels were quantified by ELISA. When nonathletes were compared with the kayakers, the latter exhibited lower plasma concentrations of IL-1**β**, IL-18, and IFN-**γ** as well as a lower concentration of IL-1ra. Positive correlations between IL-18 and B cells in the athletes were also found. These results seem to reinforce the anti-inflammatory role of regular training.

## 1. Introduction

Regular exercise has been associated with protection against mortality from all causes, primarily to protect against atherosclerosis, type 2 diabetes, colon and breast cancer [1]. In addition, exercise training is effective in treating patients with ischemic heart disease [2], heart failure [3], type 2 diabetes [4], and chronic obstructive pulmonary disease [5]. The protective effect of exercise against diseases has been attributed to the anti-inflammatory effect of exercise practiced regularly causing endocrine effects that directly mediate the antiinflammatory response of cytokines [6, 7]. Cytokines, which are bioactive polypeptides, are important in cell regulation. They are messengers that regulate and bring information to the cells of the immune system [7]. When a chronic systemic low grade inflammation occurs, proinflammatory and anti-inflammatory circulating interleukin levels are increased two- to four-fold and a small elevation in the number of circulating neutrophils and natural killer cells [8, 9] is also seen.

Prolonged exercise, on the other hand, has been associated with a transient depression in the immune system [10]. Both prolonged exercise and competitive periods are able to impair the athletes immune function. The number and functional capacity of circulating leukocytes can be reduced when repeated series of strenuous exercise are performed. The reason for this is probably related to the increased levels of stress hormones released during exercise [11]. It is also known that acute administration of steroid glucocorticoids [12] and exercise [13, 14] may cause a temporary inhibition of interferon gamma (IFN-*γ*) production. It has been suggested that this may be an important mechanism by which exercise leads to a depressed immune function [13, 14]. Our group has recently shown [15] that the frequency of monocytes and dendritic cells producing IL-1*β*, IL-12, IFN-*γ*, TNF-*α*, and MIP-1*β* decreased in highly trained swimmers throughout a training season. It has been proposed that the anti-inflammatory effects of exercise [16], possibly mediated by increased levels of IL-10 in the athletes, would be able to induce a degree of immunosuppression that could contribute to an increased susceptibility to upper respiratory tract infections during periods of intensified training. 

At rest, the immune function of athletes seems to be similar to nonathletes [10, 11]. However we need more data on the levels of circulating cytokines and leukocyte subpopulations in these two populations. The aim of this study was to investigate whether successive years of training and competition at the highest level would be able to induce long-lasting substantial differences in peripheral blood circulating cytokine levels (IL-1*β*, IL-1ra, IL-6, IL-10, IL-18, IFN-*γ*, and TNF-*α*) and leukocyte subpopulations by comparing a group of elite kayakers with age-matched nonathletes.

## 2. Materials and Methods

### 2.1. Participants Characteristics

The elite athlete sample was comprised of 13 elite kayakers, with at least 3 years of kayak training at elite level, mean age 20 ± 3 years, with an average body mass of 75.0 ± 7.9 kg, 177.3 ± 7.1 cm in height, and a VO_2max⁡_ of 58.3 ± 7.8 mL·kg^−1^·min^−1^. The control group consisted of seven healthy men who did not practiced regular physical activity and with a mean age of 18.2 ± 1.1 years, body mass of 81.3 ± 13.8 kg, and 171.9 ± 4.5 cm height. After oral and written explanation, written consent was obtained and the experimental protocol approved by the Ethics and Human Subjects Review Board at the Faculty of Sports Science and Physical Education, University of Coimbra.

### 2.2. Venous Blood Sampling

For this study, a venous blood sample of the athletes and controls was taken in November, during a specific training period where kayakers worked out with reduced loads and intensities, after an off season transitional period of six weeks, which would be the start of a new training season.

For this study, 20 mL of peripheral blood was collected by venipuncture of the antecubital vein, after 48 hours of rest from the last training session. Peripheral blood leukocytes levels, including neutrophils, lymphocytes, and monocytes, were determined using a cell counter (Beckman Coulter T66, USA).

### 2.3. Flow Cytometry

Lymphocyte subsets were determined by flow cytometry, including the total number of T lymphocytes (CD3^+^) and their T-cell subpopulations CD3^+^CD4^+^ and CD3^+^CD8^+^, B lymphocytes (CD19^+^), and natural killer cells (NK) (CD3^−^CD56^+^). Staining of the cells was performed using the LYMPHOGRAM kit (CYT-C001, Cytognos, Spain) according to the manufacturer's instructions. The stained cells were analyzed on a FACScan flow cytometer (FACSCalibur, BD, San Jose, CA, USA) using CellQuest software (BD Biosciences). Data were analyzed using the software “Infinicyt” (Cytognos, Spain).

### 2.4. Enzyme Immunoassay (ELISA)

The concentrations of cytokines IL-1, IL-1RA, IL-6, IL-10, IL-18, TNF-*α*, and IFN-*γ* in plasma were determined by sandwich ELISA kits, according to the manufacturer's instructions (Invitrogen, Nivelles, Belgium).

### 2.5. Statistical Analysis

For this study we considered the mean and standard deviation values. Because according to the Shapiro-Wilk the samples were not normally distributed, we used the Mann-Whitney *U* test to detect differences between athletes and controls and the Spearman correlation test to study the correlations between variables. The value of significance was set at *P* < 0.05. Statistical analysis was performed using the SPSS software for Mac (version 19.0).

## 3. Results

 Lower levels of IL-1*β*, IL-1ra, IL-18, and IFN-*γ* plasma concentrations were found for kayakers when compared to the nonathletes. The same was observed for the NK cell population. No differences for IL-10 and IL-6 plasma concentrations were found. The total number and percentage of leukocytes, monocytes, granulocytes, T lymphocytes, and their subpopulations and B lymphocytes did not differ between groups. A compilation of the data is presented in Table 1.

Using the Spearman test (Rho), correlations between cells (total leukocytes, lymphocytes, T lymphocytes, and subsets) and plasma interleukin concentrations were found for the kayakers. Leukocytes (WBC) positively correlated with IL-1ra (*ρ* = 0.67, *P* < 0.05) and IL-18 (*ρ* = 0.50, *P* < 0.05). Negative correlations were found between the % CD3^+^ and IFN-*γ* (*ρ* = −0.54, *P* < 0.05) and the total number of CD3^+^CD8^+^ cells and IL-1*β* (*ρ* = −0.47, *P* < 0.05). Correlations were also found between the % of B cells and IL-18 (*ρ* = 0.65, *P* < 0.05) and IFN-*γ* (*ρ* = −0.52, *P* < 0.05). The total number of B lymphocytes also correlated with IL-18 (*ρ* = 0.69, *P* < 0.05). All correlation results are shown in Table 2.

## 4. Discussion

The results of the present study show that plasma concentrations of IL-1*β*, IL-1ra, IL-18, and IFN-*γ* were lower in the kayakers compared with the nonathletes (Table 1). These results seem to confirm the anti-inflammatory effect of training [17] and consequent inhibition of IFN-*γ* production by T cells [13, 14]. Kayakers also showed slightly lower levels of TNF-*α* than controls. 

Our results showed that the concentration of IFN-*γ* in peripheral blood of athletes was positively correlated with the number of B lymphocytes and inversely correlated with circulating levels of T lymphocytes (Table 2). The mechanism of IFN-*γ* production by B lymphocytes is still under study however it is suggested that IFN-*γ* produced by B cells has an amplifying effect on the Th1 cell response [18]. The significant differences in IFN-*γ* plasma concentration between kayakers and the control group could occur due to exercise being able to cause a temporary inhibition of IFN-*γ* production by T and B lymphocytes, and it has been suggested that this may be an important mechanism by which exercise induces a depression in immune system function [13, 14].

In the present study, NK cells were lower in the kayakers than in the control group. A lower value of CD3^−^CD56^+^ cell counts, lower value of CD56^dim⁡^ subpopulation, and reduced expression of the IFN-*γ* receptor NK cell expression were also found in swimmers during a training season and could partially explain the higher frequency of upper respiratory symptoms (URS) observed during the training phases of increased volume and intensity [19]. The effect of exercise intensity in the cytolytic activity of NK cells seems to present a dual-phase effect, with initial gain followed by delayed suppression [20, 21]. Many studies support the increase in NK cell numbers in the circulation during progressive exercise, as well the fast return of NK cells towards preexercise levels during subsequent recovery [22, 23]. The alterations that occur after a set of prolonged exercises may lead to natural suppression of the NK and T cells activity, which could potentially present benefits to the transplanted patients when rejection risk is concerned [20, 21] but could increase susceptibility to URS in athletes subjected to high intensity training.

 Initially it was believed that the T helper I (Th1), CD8^+^ cytotoxic lymphocytes and NK cells were the only cell types producing IFN-*γ*. Currently there is evidence that other cells such as B cells, NKT cells, and professional antigen-presenting cells (APCs) secrete IFN-*γ* [24]. IFN-*γ* production is controlled by cytokines secreted by APCs, most notably IL-12 and IL-18. These cytokines serve as a bridge to link infection with IFN-*γ* production in the innate immune response [24, 25]. In macrophages, NK, and T cells, the combination of IL-12 and IL-18 stimulation further increases IFN-*γ* production. In our study, IL-18 concentration correlated positively with both the percentage and absolute numbers of B cells (CD3^−^CD19^+^) (Table 2). This result seems to be important to the health of athletes, because recent studies have shown that IL-18 is involved in the response and recruitment of B lymphocyte cells, specifically the activation of B cells residing in the extrafollicular plasma zone of the spleen, and IL-18 production is regulated by NK T cells (CD3^+^CD56^−^) preventing the formation of mature marginal centers [26]. In another study, with different B lymphocytes subpopulations, in normal and chronic proliferative disorders, it was suggested that a disturbed expression of IL-18 and/or of its receptor on neoplastic B cells could in certain cases facilitate the growth of tumors, as they may affect antitumor effector mechanisms because of poor regulation of IL-18 production [27]. Our results also showed that in athletes, submitted to high level intensity exercise, the plasma concentration of IL-18 was lower than in individuals who do not exercise regularly. It was postulated that exercise may lower IL-18 concentration via insulin-signaling modification [28]. A significant decrease in the concentration of IL-18 has been observed in cyclists 24 hours after completing a 230 km road race [29]. On the other hand, increased concentrations of IL-18 have been associated with several components of the metabolic syndrome and used as a factor in predicting diabetes, atherosclerosis, and cardiovascular disorders [30–32].

 In the kayakers, a significant inverse correlation between IL-1*β* and CD3^+^CD8^+^ cells was also found. The CD3^+^CD8^+^ cells are normally exposed during an inflammatory response to IL-1*β*, IL-6, and IL-23 that arise especially in ischemic lesions [33]. It has been shown that the proinflammatory cytokines determine the susceptibility of human CD8 T cells to Fas-mediated activation-induced cell death through modulation of FasL and c-FLIP(S) expression [34]. Recent findings showing that cytokines act on all lymphocytes within reactive lymph nodes during active immune responses indicate that proinflammatory cytokines produced by APCs are likely to affect the function of all reactive T cells [35]. According to Gleeson and collaborators [16], the long hours of hard training that elite athletes undertake induce an anti-inflammatory state that may cause a degree of immunosuppression that could make them more susceptible to minor infections.

## 5. Conclusion

 When comparing athletes before a training period with a control group of not physically active persons, it was observed that athletes had a lower concentration of IL-1*β*, IL-18, and IFN-*γ* that are proinflammatory cytokines. These results appear to demonstrate the anti-inflammatory effects that regular training exercise practice seems to induce and could explain the beneficial effects of long-term regular exercise, manly in preventing chronic low grade inflammation and its consequences. As stated by Gleeson et al. [16], in the athletes, an increased risk of minor upper respiratory infections may be a small price to pay for the long-term health benefits of high level exercise practice.

## Figures and Tables

**Table 1:** Peripheral blood leukocyte and plasma cytokine levels in elite kayakers and nonathletes.

	Kayakers (*N* = 13)	Controls (*N* = 7)	*P*
Total Leukocytes (10^9^/L)	9.40 ± 1.63	8.41 ± 1.88	0.24
Monocytes (10^9^/L)	0.41 ± 0.16	0.66 ± 0.69	0.23
% monocytes	4.61 ± 1.88	7.17 ± 6.63	0.55
Neutrophils (10^9^/L)	5.60 ± 1.45	5.31 ± 1.36	0.32
% neutrophils	63.0 ± 6.2	63.1 ± 5.2	0.96
Lymphocytes (10^9^/L)	2.33 ± 0.46	2.41 ± 0.69	0.41
% lymphocytes	25.2 ± 5.4	28.7 ± 6.1	0.91
CD3^+^ T cells (10^9^/L)	1.53 ± 0.47	1.76 ± 0.47	0.22
% CD3^+^ T cells	66.2 ± 16.2	74.4 ± 11.7	0.15
CD3^+^CD4^+^ T cells (10^9^/L)	0.86 ± 0.28	0.95 ± 0.28	0.35
% CD3^+^CD4^+^ T cells	56.3 ± 6.3	53.7 ± 5.9	0.22
CD3^+^CD8^+^ T cells (10^9^/L)	0.54 ± 0.18	0.63 ± 0.21	0.22
% CD3^+^CD8^+^ T cells (10^9^/L)	34.9 ± 5.5	35.5 ± 3.8	0.45
CD3^−^CD19^+^ B cells (10^9^/L)	0.40 ± 0.13	0.35 ± 0.13	0.22
% CD3^−^CD19^+^ B cells	17.2 ± 4.6	14.4 ± 4.0	0.10
CD3^−^CD56^+^ NK cells (10^9^/L)	0.06 ± 0.03	0.10 ± 0.04	0.02*
% CD3^−^CD56^+^ NK cells	2.46 ± 0.87	5.28 ± 3.31	0.02*
IL-1β pg/mL	4.61 ± 2.18	8.74 ± 3.16	0.03*
IL-1ra pg/mL	20.58 ± 7.09	78.67 ± 84.62	0.01*
IL-6 pg/mL	14.18 ± 5.83	11.33 ± 4.28	0.19
IL-10 pg/mL	16.53 ± 14.90	9.05 ± 3.52	0.20
IL-18 pg/mL	354.13 ± 83.70	1758.86 ± 1794.27	<0.01*
IFN-*γ* pg/mL	6.74 ± 5.06	13.86 ± 3.68	0.01*
TNF-*α* pg/mL	31.38 ± 33.41	35.54 ± 13.36	0.11

Values are expressed in mean and standard deviation. *P < 0.05.

**Table 2:** Spearman correlations (*ρ*) between peripheral blood leukocyte subpopulations and plasma cytokine concentrations in elite kayakers (*n* = 13).

Cells		Cytokines					
	IL-1*β* pg/mL	IL-1ra pg/mL	IL-6 pg/mL	IL-10 pg/mL	IL-18 pg/mL	IFN-*γ* pg/mL	TNF-*α* pg/mL
WBC (10^3^/µL)	0.22 (0.23)	0.67* **(<0.01)**	−0.03 (0.45)	0.03 (0.46)	0.50* **(0.04)**	0.19 (0.27)	0.08 (0.39)
% LY	−0.42 (0.07)	−0.05 (0.42)	−0.37 (0.11)	−0.20 (0.26)	−0.25 (0.20)	−0.40 (0.09)	−0.25 (0.20)
LY (10^3^/µL)	−0.18 (0.27)	0.39 (0.08)	−0.24 (0.22)	−0.15 (0.32)	0.11 (0.36)	−0.10 (0.36)	−0.09 (0.38)
% CD3^+^	−0.31 (0.14)	−0.22 (0.23)	−0.18 (0.27)	−0.16 (0.30)	−0.34 (0.12)	−0.54* **(0.03)**	−0.04 (0.44)
CD3^+^ (cell/µL)	−0.44 (0.06)	0.23 (0.22)	−0.33 (0.14)	−0.11 (0.36)	0.03 (0.45)	−0.27 (0.19)	0.14 (0.32)
% CD3^+^CD4^+^	0.30 (0.15)	0.03 (0.45)	0.12 (0.35)	−0.06 (0.41)	−0.25 (0.19)	0.12 (0.35)	0.13 (0.34)
CD3^+^CD4^+^ (cell/µL)	−0.32 (0.14)	0.21 (0.24)	−0.30 (0.16)	−0.20 (0.26)	−0.01 (0.47)	−0.35 (0.13)	0.16 (0.30)
% CD3^+^CD8^+^	−0.29 (0.16)	−0.09 (0.38)	0.01 (0.50)	0.07 (0.41)	0.31 (0.14)	−0.04 (0.49)	0.01 (0.50)
CD3^+^CD8^+^ (cell/µL)	−0.47* **(0.04)**	0.18 (0.27)	−0.11 (0.36)	0.14 (0.32)	0.12 (0.34)	−0.08 (0.39)	0.21 (0.24)
% CD3^−^CD19^+^	0.03 (0.45)	0.03 (0.45)	0.17 (0.28)	0.17 (0.29)	0.65* **(<0.01)**	0.52* **(0.04)**	0.07 (0.40)
CD3^−^CD19^+^ (cell/*µ*L)	−0.12 (0.34)	0.30 (0.15)	−0.10 (0.37)	0.05 (0.43)	0.69* **(<0.01)**	0.27 (0.19)	−0.02 (0.46)
% CD3^−^CD56^+^	−0.16 (0.29)	0.23 (0.21)	−0.17 (0.29)	−0.20 (0.26)	−0.08 (0.38)	0.01 (0.48)	0.16 (0.30)
CD3^−^CD56^+^ (cell/µL)	−0.27 (0.18)	0.38 (0.09)	−0.24 (0.22)	−0.17 (0.29)	0.08 (0.39)	−0.04 (0.44)	0.05 (0.43)

WBC: withe blood cells (leukocytes). *P < 0.05.
